# Radiologic Findings in a Patient With Mitochondrial Encephalomyopathy With Lactic Acidosis and Stroke-Like Episodes Syndrome: A Case Report

**DOI:** 10.7759/cureus.75268

**Published:** 2024-12-07

**Authors:** Sariah Watchalotone, Halley McDonald, Jessica Degolier, Alex Schlangen, Imtiaz Ahmed

**Affiliations:** 1 Radiology, College of Osteopathic Medicine, Midwestern University, Glendale, USA; 2 Anesthesiology, West Virginia University, Morgantown, USA; 3 Emergency Medicine, Central Michigan University, Mount Pleasant, USA; 4 Radiology, Tempe St. Luke's Hospital, Tempe, USA

**Keywords:** bilateral basal ganglia calcification, central venous thrombosis, melas syndrome, mitochondrial disorder, rare genetic disorder

## Abstract

Mitochondrial encephalomyopathy with lactic acidosis and stroke-like episodes (MELAS) syndrome is a rare, genetically inherited mitochondrial disorder that typically manifests in childhood. The most common radiologic features include basal ganglia calcification, atrophy, and stroke-like cortical lesions. We present the case of an 18-year-old female patient with no known medical history who arrived at the emergency department with altered mental status following a suicide attempt. The patient’s initial workup, including a computed tomography (CT) scan of the brain, revealed abnormal findings, prompting further investigation into the patient’s medical history. It was later discovered that the patient had a previous diagnosis of MELAS syndrome. This diagnosis helped explain both the radiologic abnormalities and her psychiatric symptoms. This case underscores the importance of recognizing radiologic presentations of rare conditions such as MELAS syndrome, which may contribute to the broader spectrum of clinical manifestations associated with the disease.

## Introduction

Mitochondrial encephalomyopathy with lactic acidosis and stroke-like episodes (MELAS) syndrome is a rare genetic disorder primarily associated with maternally inherited mutations. The onset typically occurs in childhood, between the ages of two and 10 years, and is characterized by progressively worsening, recurrent episodes of headaches, encephalopathy, myopathy, lactic acidosis, and stroke-like episodes [1,2]. On imaging, MELAS syndrome manifests as multifocal stroke-like cortical lesions, often exhibiting a "shifting spread pattern" across cerebral vascular territories, predominantly affecting the parieto-occipital and parietal-temporal lobes, along with basal ganglia calcification [1]. Acute stroke-like episodes may present with focal hyperperfusion, which can mimic an acute stroke and lead to inappropriate thrombolytic treatment, potentially resulting in complications such as hemorrhage [3,4]. While basal ganglia calcification is a common idiopathic finding in older patients, it should be considered pathologic in individuals younger than 40 years of age until proven otherwise [5]. Differential diagnoses for stroke-like lesions include other mitochondrial disorders, such as myoclonus epilepsy with ragged red fibers (MERRF), Leigh syndrome, Kearns-Sayre syndrome (KSS), Creutzfeldt-Jakob disease, viral encephalitis, and status epilepticus [1,5,6].

## Case presentation

**Figure 1:**
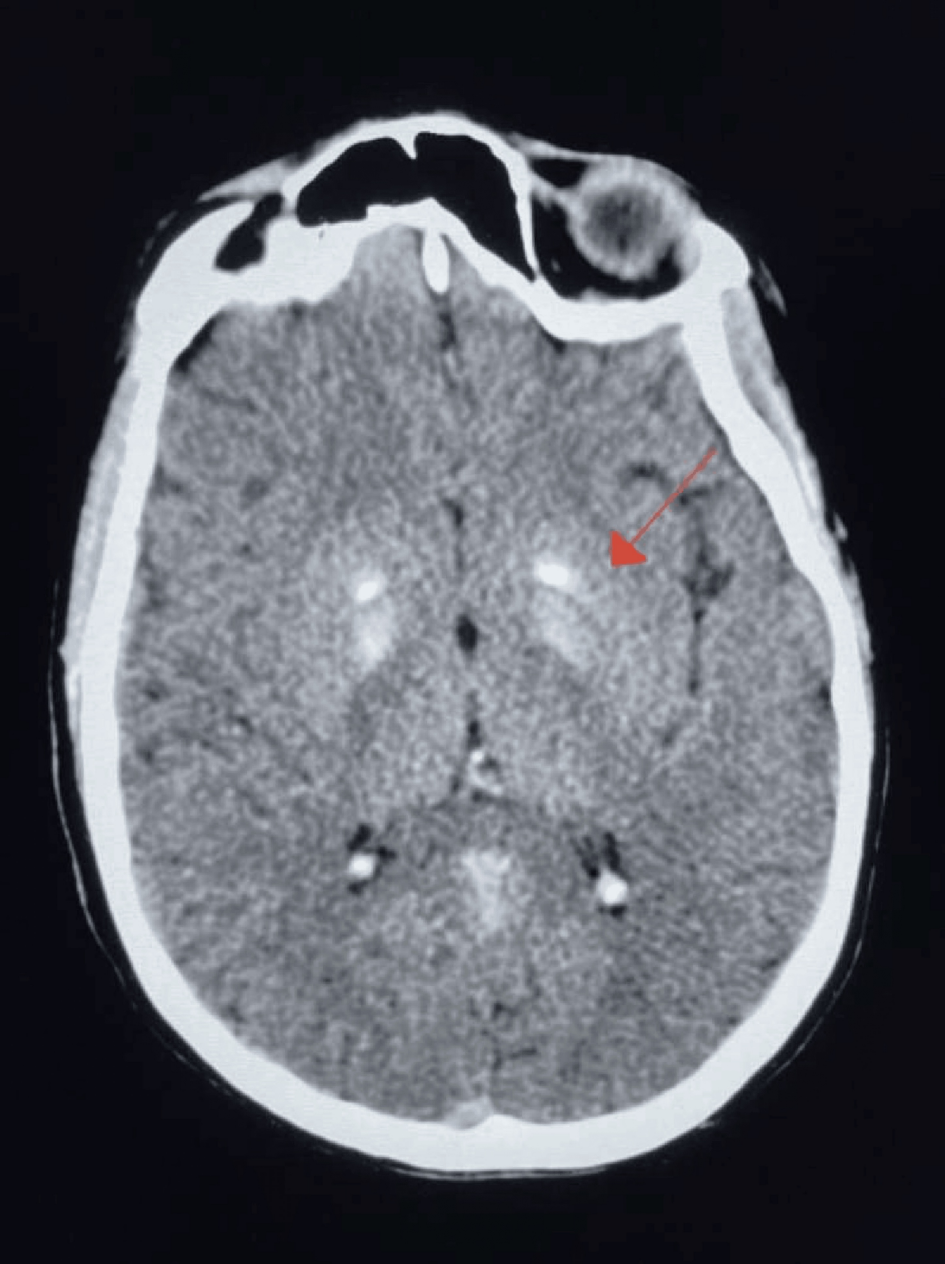
CT imaging of the head in the axial plane showing bilateral basal ganglia calcification in a tram-track-like pattern (red arrow).

**Figure 2:**
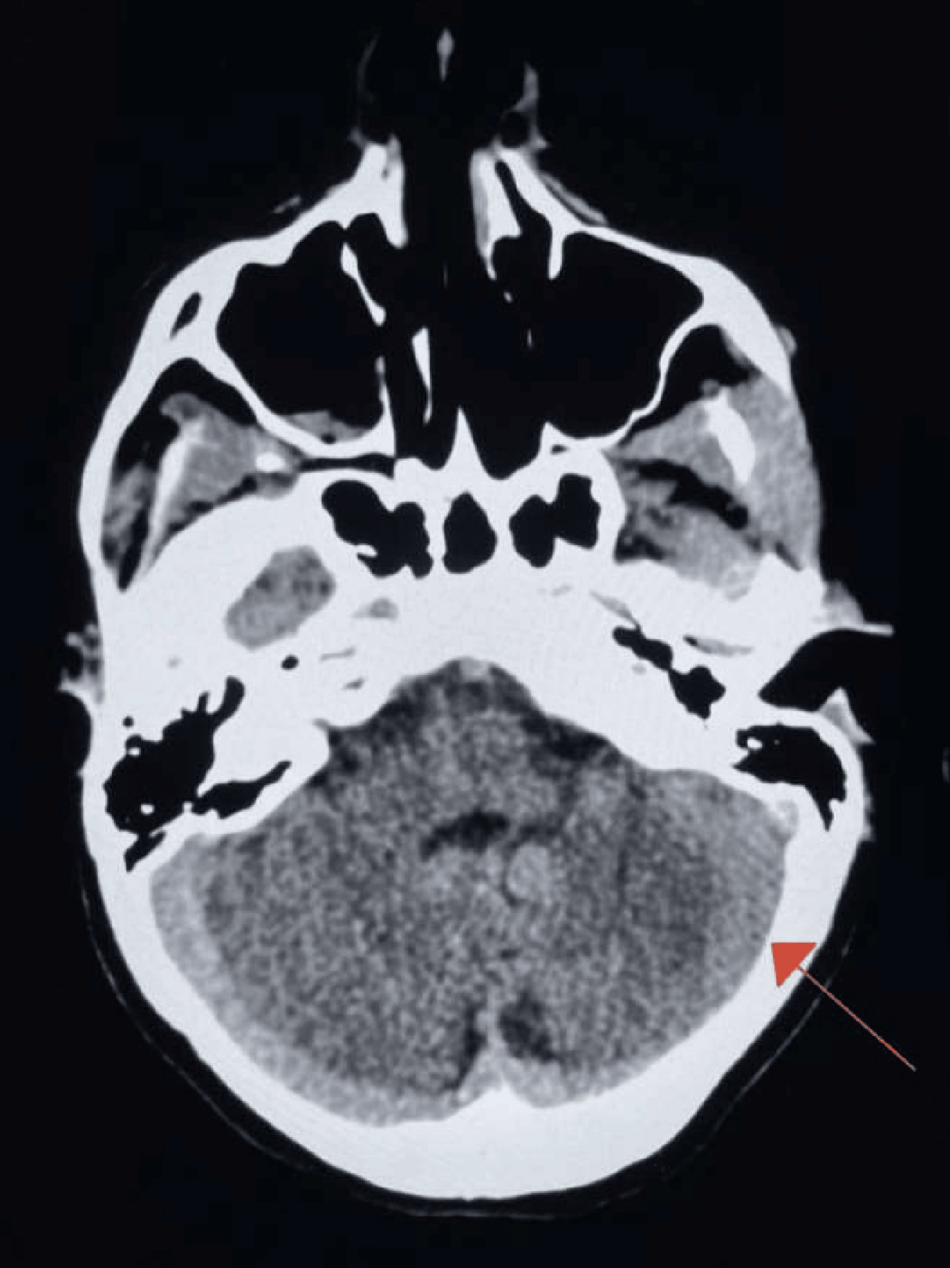
CT imaging of the head in the axial plane showing hypoattenuation of the left sigmoid sinus (red arrow). Before confirming the diagnosis of MELAS syndrome, a CT venogram was recommended for a more sensitive evaluation to rule out venous sinus thrombosis.

An 18-year-old female patient presented to the emergency department with altered mental status following a suicide attempt in which she ingested 30 Midol tablets, 13 promethazine tablets, and an unknown quantity of acetaminophen. The patient had no recollection of the incident but was found unclothed with bruises by her roommate. She reported fatigue and denied any additional symptoms, including fever, chills, nausea, vomiting, chest pain, shortness of breath, or abdominal pain. Upon arrival at the emergency department, her medical history was unknown. It was later revealed that she had a past diagnosis of MELAS syndrome confirmed by genetic testing. CT imaging of the head revealed basal ganglia calcification (Figure 1) and nasopharyngeal soft tissue prominence. Hypoattenuation of the left sigmoid sinus was also noted (Figure 2). The patient was subsequently admitted to the behavioral health unit for further evaluation and treatment.

## Discussion

MELAS syndrome is a genetically inherited mitochondrial disorder that typically presents in childhood. Mitochondrial diseases affect an estimated 1 in 5,000 people, with MELAS having an estimated incidence of 1 in 4,000 [7,8]. The most common psychiatric presentations of MELAS syndrome include mood disorders, psychosis, anxiety, and cognitive deterioration [2]. The common radiologic features of MELAS include CT findings of basal ganglia calcification, atrophy, and multiple infarcts [1]. In this case, the main CT findings were hypoattenuation of the left sigmoid sinus, bilateral basal ganglia calcification, and nasopharyngeal soft tissue prominence.

Differential diagnoses for hypoattenuation of the sigmoid sinus include central venous thrombosis, arachnoid granulation, dural sinus cyst, and dural sinus adipose tissue [9]. Due to concerns about cerebral venous thrombosis, a CT venogram was recommended for further evaluation. Central venous thrombosis can present with stroke-like symptoms and is a rare but recognized complication of MELAS syndrome [10]. This case contributes to the expanding spectrum of clinical findings associated with MELAS.

Pathological basal ganglia calcification has a broad differential diagnosis, including genetic disorders (e.g., mitochondrial diseases, Cockayne syndrome, Down syndrome, tuberous sclerosis, and Fahr’s syndrome), infectious causes (e.g., toxoplasmosis, cytomegalovirus, human immunodeficiency virus, and neurocysticercosis), metabolic disorders (e.g., parathyroid disorders), and toxic causes (e.g., lead and carbon monoxide poisoning) [11].

## Conclusions

This case highlights the importance of integrating unique radiologic findings with the patient’s clinical history. In this instance, the patient’s altered mental status, radiologic findings of basal ganglia calcification, and nasopharyngeal soft tissue prominence raised concerns for potential illicit drug use. The patient’s prior diagnosis of MELAS syndrome and her psychiatric presentation were crucial in correlating the radiologic findings with her overall clinical picture. This case emphasizes the need to recognize radiologic presentations of rare diseases like MELAS syndrome, which can broaden the spectrum of clinical findings associated with the condition.
